# Selected Technical Aspects of Molecular Allergy Diagnostics

**DOI:** 10.3390/cimb45070347

**Published:** 2023-06-29

**Authors:** Kinga Lis, Zbigniew Bartuzi

**Affiliations:** Department of Allergology, Clinical Immunology and Internal Medicine, Ludwik Rydygier Collegium Medicum in Bydgoszcz, Nicolaus Copernicus University in Toruń, 85-168 Bydgoszcz, Poland

**Keywords:** specific IgE, allergy, molecular diagnostics, allergen extract, allergen component, anti-CCD, ISAC, ALEX

## Abstract

Diagnosis of allergic diseases is a complex, multi-stage process. It often requires the use of various diagnostic tools. The in vitro diagnostics (IVD), which includes various laboratory tests, is one of the stages of this process. Standard laboratory tests include the measurement of the serum concentration of specific immunoglobulin E (sIgE) for selected allergens, full allergen extracts and/or single allergen components (molecules). The measurement of IgE sIgE to the allergen components is called molecular allergy diagnosis. During the standard laboratory diagnostic process, various models of immunochemical tests are used, which enable the measurement of sIgE for single allergens (one-parameter tests, singleplex) or IgE specific for many different allergens (multi-parameter tests, multiplex) in one test. Currently, there are many different test kits available, validated for IVD, which differ in the method type and allergen profile. The aim of the manuscript is to present various technical aspects related to modern allergy diagnostics, especially in the area of molecular allergy diagnostics.

## 1. Introduction

Allergy and hypersensitivity are increasingly becoming serious global public health problems. These reactions are based on complex pathophysiological mechanisms leading to the dysfunction of various organs and even leading to a life-threatening condition. Both allergy and hypersensitivity can develop at any age and have a significant impact on the quality of life of patients and their families [1]. The terms “hypersensitivity” and “allergy” are not synonymous. Hypersensitivity is defined as conditions clinically resembling an allergy that cause objectively reproducible signs or symptoms, initiated by exposure to a specific stimulus at a dose tolerated by the body of a healthy person. Allergy is a hypersensitivity reaction based on certain or highly probable immunological mechanisms. An allergic reaction occurs when it is triggered by allergens to which the affected person is allergic (i.e., has specific antibodies or immunologically competent cells directed against these allergens) [1]. 

**Figure 1:**
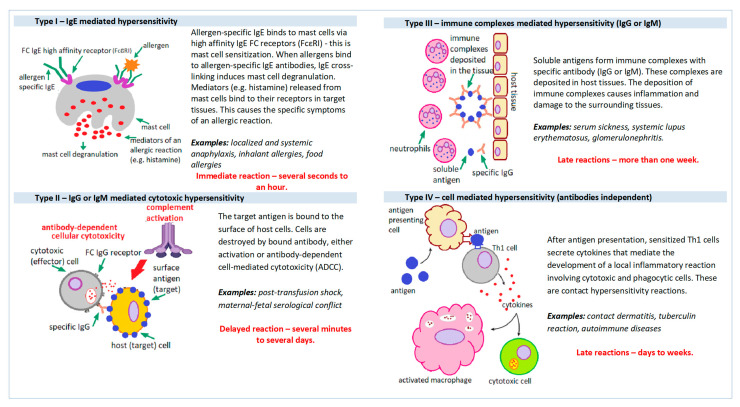
Mechanisms of hypersensitivity reactions.

The currently used division of hypersensitivity reactions was proposed in 1963 by Gell and Coombs. This classification divides hypersensitivity reactions into four distinct groups based on the mechanism of tissue damage (Figure 1): Type I (immediate, IgE mediated), Type II (cytotoxic or IgG/IgM mediated), Type III (immediate mediated by the immune complex) and type IV (late, T-cell mediated, antibody-independent) [2].

Allergy diagnosis is a complex and multi-stage process. It begins with a clinical interview, physical examination of the patient and additional tests. Additional tests used in the diagnosis of allergic diseases can be divided into in vivo tests (e.g., skin prick tests, challenge tests) and in vitro tests (laboratory tests). Allergy tests validated for IVD can in principle only confirm or rule out Type I (IgE-mediated) hypersensitivity reactions. The in vitro tests include, among others, serological diagnostics based on the measurement of serum levels of allergen-specific immunoglobulins IgE (sIgE) [3,4].

The possibilities of modern serological diagnostics of allergy are very extensive. The IgE specific for both whole allergen extracts and individual allergen components can be measured in the patient’s blood serum. It is possible to determine sIgE both for single allergens and for mixes of various allergens (so-called allergen mixes). Currently available serological tests can also be used to measure the serum sIgE level for a single allergen (monspecific tests, so-called singleplex tests), as well as for many different allergens in one test (multispecific tests, so-called multiplex tests) [5,6].

## 2. Allergy Diagnosis Based on the Measurement of Allergen-Specific IgE (sIgE)

This type of laboratory test can be performed with test kits from various manufacturers that have been validated for in vitro diagnostics (IVD) and have certificates required by dedicated legal regulations in force in a specific country. The manufacturers of the test kits guarantee that the results of laboratory tests measured using the analytical reagents produced by them are reliable and repeatable. The test kits are provided with instructions for use, which also define the conditions for the storage of reagents and the collection of biological material for testing and storage. The manual also specifies possible factors interfering with the analytical procedure and the patient’s preparation for a specific laboratory test, including the possible need to discontinue various drugs or other clinically significant parameters that may interfere with the analytical procedure.

Routinely, under standard conditions, the measurement of IgE is measured in the serum of venous blood. Blood for the test should be collected in the morning, although in the case of IgE it is not an obligatory recommendation, as this parameter is not subject to significant daily fluctuations. In these types of laboratory tests, there is also no need to stop treatment with medicines normally used to treat allergies, because they do not interfere with the analytical procedures used. The patient does not have to be fasting, although it is definitely better to fast before each laboratory test, even if it is not absolutely required. It may be important to refrain from ingestion dietary supplements, mainly vitamins (especially vitamin C and biotin), which may significantly interfere with some laboratory procedures and may cause false-negative or false-positive results. Vitamins should be stopped at least 7 days before taking blood for laboratory testing. It is important that each laboratory prepares appropriate guidelines for the patient on how to prepare for the collection of biological material for a specific laboratory test [7,8].

**Figure 2:**
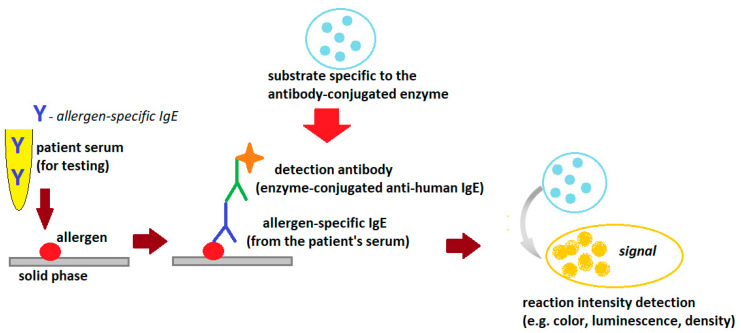
General scheme of the ELISA reaction detecting allergen-specific.

The technique of all currently available serum sIgE assays is based on the Enzyme-Linked Immunosorbent Assay (ELISA) method, which involves the binding of IgE antibodies from the patient’s serum to the allergens for which these antibodies are specific (Figure 2).

The allergy laboratory tests use standardized allergens, extracts or single allergen components (native or recombinant), which can be suspended in the liquid phase or bound in the solid phase (e.g., cellulose paper, cellulose sponge, polymers, glass, paramagnetic particles and others). If the patient’s serum contains IgE antibodies specific to the allergen used in the test, the allergen/allergen-specific IgE complexes are formed. The complexes are bound to the solid phase and unbound serum components are washed out of the reaction tube with a washing solution (e.g., phosphate buffer). These complexes are then visualized using antibodies specific to human IgE (mouse, goat or rabbit) labeled with the enzyme and a substrate compatible with the labeling enzyme used (chromogen or fluorochrome). The intensity of the reaction is expressed as color intensity or fluorescence intensity (depending on the marker used) and is proportional to the concentration of specific IgE in the tested serum. If appropriate calibration standards are used, the method can be quantitative. Quantitative or semi-quantitative methods are usually used to detect sIgE [5,9,10].

The solid-phase immunoassays based on the ELISA model for routine immunochemical diagnostics, including the measurement of specific IgE levels, were available as early as the 1970s. Initially, radioimmunoassays (radioallergosorbent test, RAST) were used. RAST tests consisted of immobilizing allergen extracts on activated paper discs to bind specific IgE from the patient’s serum. Paper discs were the solid phase of the RAST test. Radiolabels were used to detect allergen/sIgE immune complexes in RAST tests. Currently, radioactive tracers are not used, mainly due to their harmfulness. They have been effectively replaced by colorimetric markers, and above all by fluorescent markers (chemiluminescent and electrochemiluminescent techniques) [5,10]. 

The materials of the solid phase of testing have also changed. In addition to paper discs, which are rarely used anymore, various types of polymers and multidimensional cellulose matrices (cellulose sponges) are more often used. There are also reaction models based on allergens suspended in the liquid phase. In this assay model, the first step of the reaction (formation of allergen/sIgE complexes) takes place in the liquid phase. In the next step, the formed allergen/sIgE complexes are immobilized on a solid phase matrix. Matrices made of glass fibers or nanoparticles with paramagnetic properties are most often used [11,12].

Qualitative, semi-quantitative and quantitative tests are used to measure the concentration of specific IgE in allergy diagnostics. Quantitative immunoassays for sIgE antibodies require the inclusion of a standard curve. Calibrators for both specific and tIgE measurements should be traceable to the World Health Organization (WHO) International Reference Preparation for Human IgE, 75/502 [10].

### Extract-Based and Component (Molecular) Immunological Allergy Diagnostic

**Figure 3:**
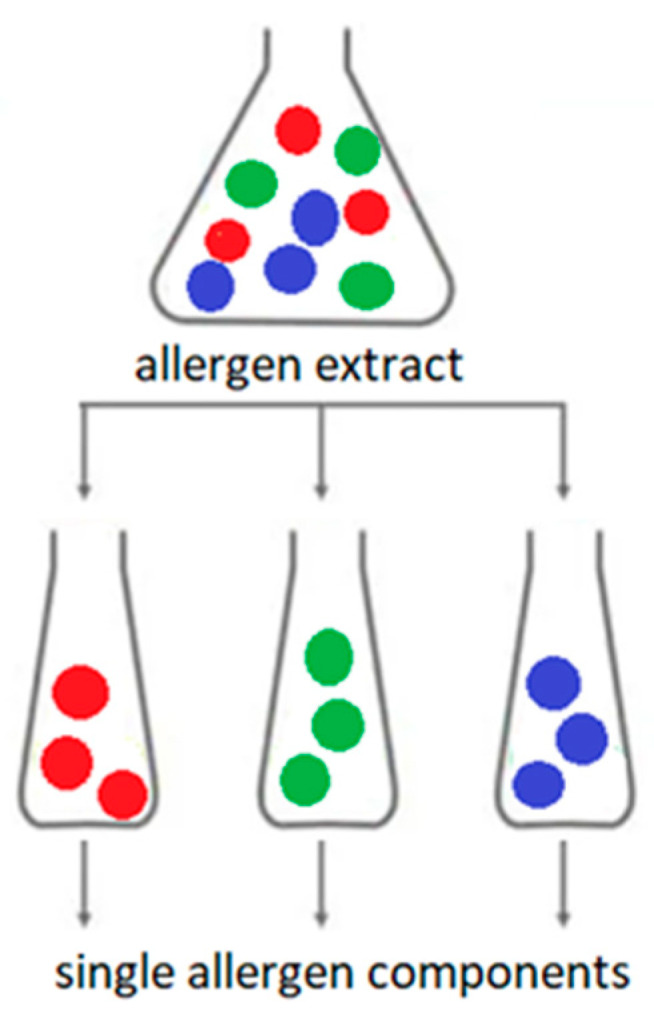
Allergen extract versus allergen components; The colored dots are individual allergen molecules.

Traditional immunological (or serological) diagnosis of allergies is based on the determination of IgE antibodies specific for extracts of native allergens. This type of allergy diagnosis is called allergy diagnosis based on extracts. Allergen extract is a mixture of allergenic and non-allergenic proteins derived from a specific allergenic source (Figure 3). 

Extracts from natural allergen sources are often heterogeneous and may contain many non-allergenic molecules. Extracts of the same allergen from different lots may differ in the composition and amount of allergenic proteins. The detailed composition of full extracts is not clearly possible to determine. Extracts from the same allergen source from different manufacturers may have different component compositions. The composition of extracts from food allergens also depends on the method and conditions of production of a particular food, cultivation and storage of plants or animal husbandry technology. In addition, the presence of bioactive molecules (e.g., proteolytic enzymes) in natural extracts may affect the stability of the allergen extract. Contamination of the natural allergen extract with proteins from other sources is also possible [13,14,15,16,17,18,19]. 

Molecular diagnosis of allergy is based on the detection in the blood serum of IgE specific against single allergen components (molecules) from specific allergen source. An allergenic component is a single protein or, less commonly, a carbohydrate moiety that can induce an allergic immune response [17,20,21,22]. In the molecular diagnosis of allergy, native or recombinant allergen components are used. Native components are obtained directly from the natural allergen extract using various physicochemical or biochemical techniques. Recombinant components are produced by genetic engineering. Recombinant molecules produced in this way are synthesized by a different organism than the one from which they originate. *Escherichia coli* (*E. coli*)*,* or yeast cells, are most commonly used for the production of recombinant allergens. This technology makes it possible to produce unlimited amounts of perfectly purified allergens with precisely defined molecular structures. The downside of recombination is that proteins produced in this way do not undergo post-translational modifications (e.g., glycosylation). This may change their spatial structure and immunological specificity. Such molecules may not bind antibodies produced by immunization with natural proteins. In turn, the advantage of recombinant allergenic components is that as they are devoid of carbohydrate groups, they do not bind clinically insignificant antibodies specific for cross-reactive carbohydrate determinants (anti-CCD). This prevents false-positive results with sIgE tests [13,14,15,16].

Traditional in vitro allergy diagnosis based on allergen extracts makes it possible to determine the source of sensitizing allergens, but it does not allow for the identification of cross-reactions or estimation of the severity of a possible reaction after contact with this allergen. This is especially important for patients allergic to food allergens. Molecular diagnosis of allergy enables accurate identification of the allergen molecule to which the patient is allergic. This gives the opportunity to precisely identify a harmful allergen, determine possible cross-reactions, predict the severity of a possible reaction after contact with this allergen, including severe anaphylactic reactions, or estimate the expected effectiveness of allergen immunotherapy. It also helps to determine the dietary management of a patient with food allergy [17,20,21].

## 3. Molecular Allergy Diagnostic-Technical Aspects and Current Possibilities

Molecular diagnosis of allergies is a method aimed at determining the individual allergic profile of the patient. It is treated as a type of personalized medicine. Currently, this type of diagnostics can be performed in two ways—by measuring the concentration of IgE specific for single allergen molecules (single-component diagnostics) or by simultaneously measuring the concentration of sIgE for many allergenic components in one test (multi-component diagnostics, multiplex) [5,6].

### 3.1. Singleplex (Monocomponent) Molecular Allergy Diagnostic

Monocomponent molecular allergy diagnostics is a measurement of the serum concentration of IgE specific for a selected allergen molecule. Validated kits from various manufacturers are available in routine laboratory practice. They differ in analytical sensitivity and specificity. These differences result from the technical and methodical construction of the test. These are optional solid or liquid phase tests. Solid phase tests may differ in the material from which the solid phase is made (e.g., cellulose, polymer or paramagnetic molecule). Native or recombinant allergen components may be used in these tests. There may be a different type of detection antibody, a different label, and a different way of detecting the result of the reaction. Each test kit is dedicated to performing the test on a compatible immunochemistry analyzer. The result obtained in singleplex studies is always a quantitative result. These techniques require a relatively large volume of serum per allergen per test (40–50 µL of serum per allergen plus an additional dead volume of approximately 100 µL). Singleplex tests are also relatively expensive per single sIgE result [5].

### 3.2. Multiplex (Multicomponent) Molecular Allergy Diagnostic

Multicomponent molecular allergy diagnostics is based on the determination of specific IgE concentration for many different allergen components in one test. Such a diagnostic model is possible thanks to the use of multi-parameter tests for in vitro diagnostics, the construction of which is based on allergen matrices and nanotechnology. Allergen matrices used in this type of analysis are a powerful diagnostic tool that enables simultaneous analysis of IgE specific for many different allergens in biological material during one analytical process. Microarray technology allows for the simultaneous analysis of thousands of parameters in a single experiment. Microspots of capture molecules, e.g., allergens, are immobilized in precisely defined places on a solid substrate (glass, plastic, cellulose plates) and then exposed to samples of the test biological material. If there are appropriate analytes in the tested biological material, they are bound to molecules coated on the matrix. The immune complexes that are the product of the reaction are then detected by appropriate immunochemical techniques, and signal detection can be based on fluorescence, chemiluminescence, mass spectrometry, densitometry or electrochemistry [23].

It is worth noting that any test in which sIgE is determined for more than one allergen at the same time is a multiparameter test. Using currently available allergy diagnostic multiplex platforms, it is possible to determine the concentration or level of allergen-specific IgE for several to several hundred allergen molecules during a single analysis. These are solid phase tests giving quantitative or semi-quantitative results. Several component or component-extract panels are available, requiring about 100–200 µL of blood serum, which are usually targeted at a specific allergen source (e.g., milk panel—contains the main allergen components of cow’s milk) and panels in which we simultaneously determine sIgE for several hundred allergen components, or both extracts and allergen components, aimed at determining the individual allergy profile of the tested patient.

The laboratory analytical platforms enabling the simultaneous detection of IgE specific for several hundred allergens are usually used in the diagnosis of multi-sensitized patients. These tests contain allergen components from several dozen different allergen sources. They can be used to detect either sIgE only for allergen components (ISAC test, ThermoFisher Scientific, Waltham, MA, USA) or sIgE for allergen components, allergen extracts and the total concentration of IgE in the blood serum (ALEX test, Macroarray Diagnostics, Vienna, Austria). Currently, two multiplex platforms, ISAC and ALEX tests, are available for broad-profile allergy molecular diagnostics. The ISAC and ALEX tests differ in the allergen profile, immunochemical technology, laboratory procedure, method of reporting results and the volume of serum needed to perform the test.

#### 3.2.1. ISAC (Immuno Solid-Phase Allergen Chip; ThermoFisher Scientific, Waltham, MA, USA)

The ISAC test is a solid phase enzyme immunoassay based on biochip technology. A biochip (microarray) is a solid matrix (e.g., a glass or plastic plate) on which specific biological molecules have been precisely applied in a specific location. This technology makes it possible to simultaneously detect the presence of many biological components in a small amount of test material (e. g. blood serum), using a small volume of reagent. In the ISAC test, allergen components applied in triplets on a glass plate (slide) bind IgE antibodies specific to them from the patient’s blood serum. Specific IgE from the test serum are captured by the allergens coated on the slide and bound to the solid surface of the test. The formed allergen/sIgE immune complexes are then detected with fluorochrome labeled anti-human IgE antibodies. Each ISAC slide contains four microarrays for four patients. The fluorescence intensity is measured with a biochip scanner that is compatible with the ISAC test slides and the fluorochrome used. The manufacturer of the ISAC test recommends the use of laser devices for confocal scanning, in particular the CapitalBio LuxScan 10 k microarray scanner (CapitalBio, San Diego, CA, USA). The microarray images are then analyzed using Phadia Microarray Image (MIA) software.

**Figure 4:**
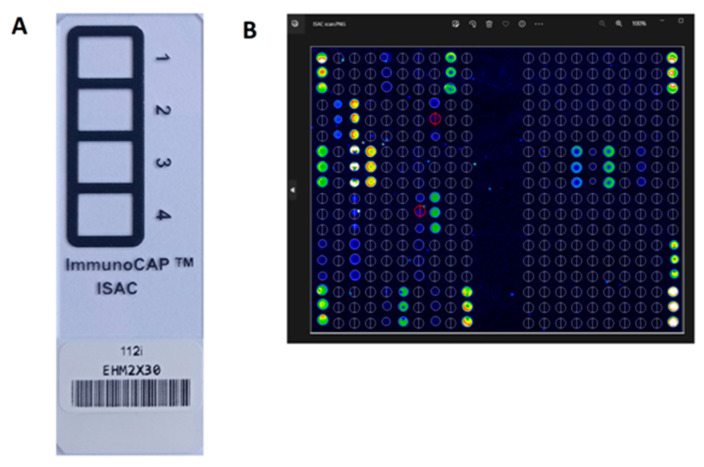
The biochip of the ISACE112i test (**A**) and scan of the biochip field for a single patient, after reaction with serum, detected in a scanner (LuxScan) with MIA software (**B**).

Figure 4 shows the ISACE112i glass biochip and single patient microarray image after reaction with the sIgE positive serum from the CapitalBio LuxScan scanner with MIA software.

The ISACE112i platform is a semi-quantitative test and sIgE antibody results are reported in ISAC Standardized Units (ISU-E) and are divided into four categories based on level: <0.3 ISU-E (undetectable); 0.3–0.9 ISU-E (low); 1–14.9 ISU-E (medium/high); >15 ISU-E (very high). The test requires 30 µL of serum or heparinized plasma. The patient does not have to stop taking any medications and does not have to fast [24]. The laboratory procedure of the ISAC test takes 4 h. Interpretation of the result can be supported by the integrated Xplain^®^ software (ThermoFisher Scientific Inc.) and the AllerGenius^®^ expert system (ARMIA, Genova, Italy) [25,26].

The first multi-parameter allergen chips of this type (ISAC test) were made available in 2001. Since 2006/2007, the ISAC test has been widely available for IVD [27,28]. The current version of this test (ISACE112i), available from 2019, enables the simultaneous determination of sIgE for 112 different single molecules from 48 different sources of plant and animal allergens (Table 1).

#### 3.2.2. ALEX (ALLERGY XPLORER, MacroArray Diagnostics GmbH (MADx), Vienna, Austria)

The ALEX test is a solid-phase enzyme immunoassay based on technology using nanoparticles (nanospheres) as allergen carriers. It is the newest multiplex in vitro allergy test currently available. The use of nanoparticle technology as carriers of allergens makes the ALEX test a test with an easily modifiable allergen profile. This makes it possible to replace one allergen molecule with another in subsequent generations of the test. Thanks to this functionality, the allergen profile of the ALEX test dynamically responds to new clinical requirements and the discovery of new relevant allergen molecules [29].

Technologies that use nanoparticles are nowadays often used in various branches of medicine, both therapy and diagnosis. Drugs and diagnostic tests using this technology are part of the trend of personalized medicine. The nanoparticles are defined as particles with at least one cross-sectional dimension in the range of 1 to 100 nm. They can be formed naturally (as a result of erosion of rocks or organic compounds), or they can be by-products of human activity, or they can be intentionally created nanoparticles of a defined size and structure (so-called engineered nanoparticles). Engineering nanoparticles can be made of various materials: gold, silver, platinum, carbon, cobalt, copper, silicon and other materials [30,31,32].

A characteristic feature of nanoparticles is a high ratio of surface area to particle volume (6 × 10^8^ m^−1^ for nanoparticles with a diameter of 10 nm), thanks to which large amounts of various substances can be placed on a small nanoparticle surface. This ratio is greater the smaller the diameter of the nanoparticle. In addition, the small dimensions of nanoparticles make it possible to place a large number of them on a small surface area of the solid phase. Depending on the material from which they were made, nanoparticles have the ability to bind various biological molecules (e.g., allergens) on their surface, thanks to which they become their carriers. The process of producing particles with desired physical, chemical or biological properties is called functionalization of nanoparticles. Functionalization affects the ability of nanoparticles to interact with specific chemical or biological substances [30,31,32].

The nanoparticles, which are carriers of various biomolecules (e.g., allergens), can be immobilized in a solid phase (e.g., on a specific reaction matrix). This enables the construction of comprehensive solid phase assays for IVD. With the use of nanoparticles, complex test platforms are constructed, which are used for simultaneous multi-analysis of a small volume of biological material (e.g., patient’s blood serum). Functionalizing biomolecules (e.g., allergens) are applied to the surface of the nanoparticles before immobilizing them in the solid phase. Such a strategy allows the use of the most optimal conditions necessary for the application of functionalizing agents to the surface of the nanosphere. Thanks to this property, the surface of the nanoparticle is used to the maximum, and the concentration of functional biomolecules (e.g., allergens) on the nanosphere is very high. Tests constructed in this way are usually characterized by very high analytical sensitivity. From the point of view of the possibility of obtaining the highest possible analytical sensitivity of the test, it is also important that the nanoparticles are coated with various functionalizing factors (e.g., allergens) independently of each other. This allows for adjusting the conditions of functionalization of nanospheres to the specific requirements of specific functional biomolecules. Thanks to such a strategy, optimal use of the surface of the nanoparticle is obtained in relation to each applied functionalizing agent. For example, in the context of tests for the analysis of sIgE, the conjugation of allergens with nanospheres is performed using protocols optimized for specific allergens taking into account their biochemical properties. In addition, the availability of relevant epitopes is controlled, which increases the detection efficiency of sIgE. During the construction of the test, previously functionalized nanospheres are bound to the solid phase matrix. Thanks to this strategy, tests with very high analytical sensitivity are obtained for each analyte independently. In addition, the small dimensions of the nanoparticles make it possible to obtain a high density of these carriers on a small area of the test matrix, which also increases the efficiency of the test. In relation to tests for the detection of sIgE, this means the possibility of constructing analytical platforms on which even several hundred different allergens are bound to the matrix. Such multiplex matrices allow for the detection of IgE specific for several hundred different allergens simultaneously (in one test) and in a small volume of blood serum. In conclusion, the analytical tests constructed in the technology of nanoparticles are characterized by very high efficiency and high analytical sensitivity independently for each measured analyte [30,31,32,33]. Currently, the ALEX test is the only multiplex analytical platform for in vitro allergy diagnostics constructed using nanoparticle technology. The FABER test (patient-friendly Allergen Nano-Bead Array; Centri Associati di Allergologia Molecolare CAAM, Roma, Italy) was also constructed using nanoparticle technology, which is currently no longer available.

**Figure 5:**
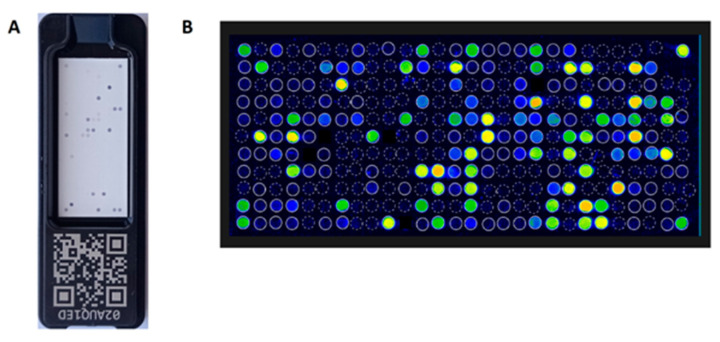
The ALEX2 biochip/cassette with positive reaction (**A**) and cartridge scan image with positive reaction in scanner (ImageXplorer) with MADx Raptor Software (**B**) author’s own photo.

Figure 5 shows the biochip/cassette of the ALEX2 test after reaction with positive patient serum and the image after scanning the cartridge with a positive reaction in the scanner (ImageXplorer, Vienna, Austria) with MADx Raptor Software.

The first generation of the ALEX test was introduced for IVD in 2017 and enabled the simultaneous measurement of sIgE levels for 282 allergens, including 126 allergen components and 156 allergen extracts, and total IgE (tIgE) concentration. The current generation of the ALEX test (called ALEX2 to distinguish it) has been available since 2019. This edition offers simultaneous measurement of sIgE concentrations for 295 allergens, including 178 molecules and 117 extracts, from most families of inhalant allergens and cross-reactive food allergens (Table 1). An important feature of the ALEX2 test is still the ability to simultaneously measure the concentration of both sIgE for extracts and allergen components, as well as the concentration of tIgE in the blood serum. The ALEX2 multiplex assay platform is a quantitative assay for sIgE and semi-quantitative for tIgE. The measured IgE concentration is expressed in kUA/L for sIgE and kU/L for tIgE. The ALEX2 test range for sIgE is 0.3–50 kU/L, and 1–2500 kU/L for tIgE. The concentration of s IgE is classified into five classes (0–4), depending on its level, respectively: class 0 (<0.3 kUA/L; negative or borderline), class 1 (0.3–1 kUA/L; low), class 2 (1–5 kUA/L; medium concentration), class 3 (5–15 kUA/L; high concentration) and class 4 (>15 kUA/L; very high concentration). Serum or plasma (except EDTA plasma) in a volume of 100 µL is required for testing. The patient does not have to stop taking any medications and fast before taking blood for analysis. The analytical procedure of the ALEX2 test takes 3.5 h [27,28,29,33]. 

It is widely believed that one of the main added values of the ALEX2 test is the ability to simultaneously measure the concentration of sIgE for whole allergen extracts, as well as for allergen particles from many food and airborne sources and tIgE. This gives the possibility of comprehensive, broad-profile in vitro diagnostics of IgE-mediated hypersensitivity reactions [34].

Another unique feature of the ALEX2 multiplex assay is that an inhibitor of antibodies against cross-reacting carbohydrate determinants (CCD) was used in the analytical procedure. CCDs are carbohydrate residues of glycoproteins, absent in mammalian proteins, that can induce the synthesis of CCD-sIgE antibodies. Anti-CCD antibodies specifically bind to CCDs that may be present on allergen particles of natural origin but are unable to induce mast cell degranulation. In most cases, they do not contribute to the clinical symptoms of allergic disease and do not give positive results in skin tests. In general, anti-CCD IgE antibodies are considered clinically insignificant (with the exception of sensitization to α-1,3-galactose-containing glycoproteins (alphaGAL) found, for example, in tick saliva), but may cause false positive results of sIgE in in vitro allergy tests. This significantly hinders the clinical interpretation of the laboratory test result, which is not adequate to the clinical manifestation of allergy observed in the patient. It is estimated that this problem may affect up to 30% of patients. The source of CCDs stimulating the synthesis of specific anti-CCD antibodies in the IgE class are most often allergens of plant pollens and venoms of Hymenoptera insects and some allergens of dust mites. Blocking anti-CCD antibodies in the standard ALEX2 assay procedure significantly reduces the number of false positive sIgE results [35,36,37,38,39].

Two methods are most commonly used to solve the problem of anti-CCD antibodies. One of these strategies is the use of recombinant allergens in the test. Recombinant allergens do not undergo natural processes of post-translational processing, e.g., glycosylation. Therefore, they do not expose carbohydrate determinants in their molecules (they are CCD-free). The second strategy is the binding of anti-CCD antibodies, which may be present in the test serum, before the actual analytical procedure (pretreatment) or during the first incubation of the test, using an anti-CCD blocker/inhibitor. For this purpose, the test serum is mixed with an excess of glycoconjugates competing for the binding of anti-CCD antibodies (this is the so-called anti-CCD blocker or inhibitor) with the appropriate volume ratio (according to the procedure). The blocking reagent usually contains a mixture of CCD molecules of natural or synthetic origin that bind specific antibodies to each other. In the initial phase of the test, anti-CCD IgE antibodies are bound to free CCDs from the CCD-blocker/inhibitor (anti-CCD CCD/IgE complexes are formed) and then they are removed from the test plate in the subsequent washing steps provided for in the analytical procedure. The CCDs inhibitor must be engineered to block only anti-CCD antibodies and not affect other sIgE antibodies. Therefore, although theoretically it would be possible to use any glycoproteins of plant origin as an anti-CCD-blocker/inhibitor, in practice, synthetic CCDs turned out to be a much better solution [40,41]. A synthetic anti-CCD antibody blocker (ProGlycAn CCD-Blocker) was used in the ALEX2 test. It is a highly purified bromelain glycoprotein that has been partially proteolized. Thanks to this enzymatic treatment, it contains no more than 4 amino acid residues. The bromelain glycoprotein prepared in this way is conjugated with human serum albumin as its carrier. The blocker/inhibitor constructed in this way binds only IgE antibodies specific for CCDs [42]. The ALEX2 standard analytical procedure begins with the incubation of the test serum with the anti-CCD blocker/inhibitor directly on the assay matrix. This procedure blocks anti-CCD IgE antibodies with 85% efficiency. The blocking efficiency of anti-CCD antibodies can be increased up to 95% by adding a 30-min incubation of the sample with the blocking reagent prior to the actual ALEX2 assay procedure [43].

After the analytical procedure is completed, the ALEX2 cartridge is scanned using a dedicated image analyzer (ImageXplorer) and the resulting image is analyzed by dedicated analytical software (MADx Raptor Software). The software calculates the concentration of sIgE bound by the individual allergens on the ALEX2 assay matrix and evaluates the level of tIgE relative to the control value [30]. The ALEX2 test result report is supplemented with a brief description of the biochemical and physicochemical properties of the allergen components for which the presence of sIgE antibodies was found and the clinical significance of the detected allergies. It is a useful tool to support the clinical interpretation of the ALEX2 test results and the planning of further therapeutic strategy.

## 4. Summary, Conclusions and Future Perspectives

Allergy diagnosis based on allergen molecules (molecular allergology; MA) is a precise tool for in vitro allergy diagnosis. The development of tools for MA significantly improved the quality of allergy diagnosis, giving the opportunity to distinguish true allergies from cross-reactions, selection of specific allergen immunotherapy and risk stratification in food allergy. Extensive, multi-parameter platforms for molecular diagnostics of allergies are precise tools for determining the individual profile of patients’ sensitization. These multiplexes are specific diagnostic tools in line with the current trend of personalized medicine [4,44]. Currently, we have a wide range of allergen components available for single-component diagnostics and tests for multi-component in vitro allergy diagnostics. Two wide-profile, multi-parameter diagnostic platforms are currently available, the ISACE112i test and the ALEX2 test. The semi-quantitative ISACE112i test simultaneously measures the level of IgE specific for allergen components, while the quantitative ALEX2 test measures the concentration of IgE specific for allergen components, allergen extracts and tIgE concentration in one test. The combination of diagnostics based on full extracts with component diagnostics additionally supplemented with tIgE concentration seems to be of great importance in the individualized diagnosis of an allergic patient, allowing for the creation of a full sensitization profile and the development of a therapeutic plan that meets the individual needs of the patient. It also seems that the use of molecular allergy diagnostics allows us to effectively optimize the costs of the diagnostic and therapeutic process [45,46].

Molecular diagnosis of allergies is undoubtedly a very valuable tool necessary in the diagnostic and therapeutic process of allergic patients, especially in difficult, life-threatening conditions or requiring an individualized dietary approach. However, it is still an insufficient tool in particularly difficult cases of allergies, e.g., to very rare allergens or in multi-allergic patients requiring personalized therapeutic strategies. It seems that in the future it will be necessary to use even more advanced diagnostic tools. In vitro diagnostics based on sIgE for epitopes of allergen molecules seem to be a promising tool for the future of allergological diagnostics [47,48].

According to Alessandri et al. [29], an ideal in vitro allergy diagnostic system should contain all the allergenic proteins to which the patient may be exposed. Each allergen component should contain all epitopes that can be recognized by sIgE. In addition, during the analytical procedure, these molecules should be accessible from all sides to allow all epitopes of a particular antigen to react with specific antibodies. In addition, each system should be easily modifiable and free from factors that cause both false positive and false negative reactions. Although in reality it is unfortunately impossible to achieve such an ideal condition, the currently available multiplex platforms for molecular diagnostics of allergies seem to meet these assumptions to a large extent.

## Figures and Tables

**Table 1:** Summary of important parameters of the ISACE112i test and the ALEX2 test.

	ISACE112i Immuno Solid-Phase Allergen Chip	ALEX2 Allergy Xplorer
The number of allergens on the test matrix	112	296
The number of allergen molecules	112	177
Number of allergen extracts	0	119
Number of recombinant molecules	74	125
Number of native molecules	38	52
Method of sIgE determination	Semi-quantitative	Quantitative
test result reporting unit	ISU-E	kU/L
Total IgE determination	No	1–2500 kU/L
CCD blocking	No	yes
CCD detection	Yes	yes
Serum volume needed for testing	30 µL	100 µL
